# Assessing the Relationship between Vector Indices and Dengue Transmission: A Systematic Review of the Evidence

**DOI:** 10.1371/journal.pntd.0002848

**Published:** 2014-05-08

**Authors:** Leigh R. Bowman, Silvia Runge-Ranzinger, P. J. McCall

**Affiliations:** 1 Liverpool School of Tropical Medicine, Liverpool, United Kingdom; 2 The Special Programme for Research and Training in Tropical Diseases of the World Health Organization (WHO/TDR), Geneva, Switzerland; University of California, Davis, United States of America

## Abstract

**Background:**

Despite doubts about methods used and the association between vector density and dengue transmission, routine sampling of mosquito vector populations is common in dengue-endemic countries worldwide. This study examined the evidence from published studies for the existence of any quantitative relationship between vector indices and dengue cases.

**Methodology/Principal Findings:**

From a total of 1205 papers identified in database searches following Cochrane and PRISMA Group guidelines, 18 were included for review. Eligibility criteria included 3-month study duration and dengue case confirmation by WHO case definition and/or serology.

A range of designs were seen, particularly in spatial sampling and analyses, and all but 3 were classed as weak study designs. Eleven of eighteen studies generated *Stegomyia* indices from combined larval and pupal data. Adult vector data were reported in only three studies. Of thirteen studies that investigated associations between vector indices and dengue cases, 4 reported positive correlations, 4 found no correlation and 5 reported ambiguous or inconclusive associations. Six out of 7 studies that measured Breteau Indices reported dengue transmission at levels below the currently accepted threshold of 5.

**Conclusions/Significance:**

There was little evidence of quantifiable associations between vector indices and dengue transmission that could reliably be used for outbreak prediction. This review highlighted the need for standardized sampling protocols that adequately consider dengue spatial heterogeneity. Recommendations for more appropriately designed studies include: standardized study design to elucidate the relationship between vector abundance and dengue transmission; adult mosquito sampling should be routine; single values of Breteau or other indices are not reliable universal dengue transmission thresholds; better knowledge of vector ecology is required.

## Author Summary

Routine sampling of mosquito vector populations is common in dengue-endemic countries worldwide despite doubts about methods used or the correlation between vector density and dengue transmission. This systematic review examined the published evidence investigating associations between vector indices and dengue cases. From a total of 1205 papers identified in database searches, 18 were included for review. A range of designs were seen, particularly in spatial sampling and analyses, and all but 3 were classed as weak study designs. Thirteen studies investigated associations between vector indices and dengue cases: 4 reported positive correlations, 4 found no correlation and 5 reported ambiguous/unreliable associations. Of 7 studies that measured the Breteau Index, 6 reported dengue transmission at levels below the currently accepted threshold of 5. There was little evidence of quantifiable associations between vector indices and dengue transmission that could reliably be used to predict outbreaks. Furthermore, appropriately designed studies are required to elucidate the relationship between vector abundance and dengue transmission. Recommendations include: standardizing study designs, particularly with respect to spatial heterogeneity; vector surveillance programs should sample adult mosquitoes; global values of the Breteau Index are not reliable universal dengue transmission thresholds; and better knowledge of vector ecology is required.

## Introduction

Global dengue incidence has increased markedly over the past 50 years to the point where it is now the most widespread mosquito-borne arboviral disease. The World Health Organisation (WHO) has estimated that 50–100 million dengue infections occur annually, while a recent study calculated that the true figure may be closer to 400 million [1]–[3]. Dengue is endemic throughout the tropics, and almost half of the world's population are at risk of infection, 75% of whom live in the Asia-Pacific region [4]. Dengue has been confirmed in 128 countries worldwide [4], [5] with major social and economic consequences [6]–[10].

Dengue is transmitted by *Aedes* mosquitoes, primarily by the highly urban-adapted vector *Aedes aegypti*, and a secondary vector *Aedes albopictus* [11]. *Ae. aegypti* thrives in the man-made urban environment, particularly in deprived communities where water storage is routine, sanitation is poor and non-biodegradable containers accumulate.

The abundance of dengue vectors species as well as dengue transmission generally show seasonal variation. Depending on the local ecology, these patterns can be in part driven by meteorological parameters such as rainfall and temperature [12], [13]. Vector surveillance is recommended by WHO and is a routine practice in many dengue-endemic countries to provide a quantifiable measure of fluctuations in magnitude and geographical distribution of dengue vector populations, ultimately with the purpose of predicting outbreaks and evaluating control [14]. The standard protocol relies on the *Stegomyia* indices, which sample the immature mosquito stages (larvae and pupae) alone [15]. This approach was developed over 90 years ago [16] for yellow fever, a markedly different infection (zoonotic in origin though ultimately transmitted between humans by *Ae. aegypti*) during a very different era (*i.e.* in terms of urbanization levels and human population densities). Focks (2004) questioned the reliability and sensitivity of the *Stegomyia* indices because they correlate poorly with abundance of adult mosquitoes, (*i.e.* the actual vector stage) which should be sampled directly [15]. Focks and others recommended sampling adult mosquitoes directly or indirectly via pupal/demographic surveys (calculating a pupae per person/area index, defined as the number of pupae divided by the number of residents/area surveyed) [15], [17]. Indices based on actual counts of adult female *Ae. aegypti* infesting houses are likely to be the most accurate, but this is rarely done [15].

The *Stegomyia* indices remain central to the monitoring of dengue vector populations. The most commonly used indices are the House (or ‘premise’) index (HI - percentage of houses infested with larvae and/or pupae;) the Container index (CI - percentage of water-holding containers infested with larvae and/or pupae) and the Breteau index (BI - number of positive containers per 100 houses inspected) [14]. Variations in sampling protocols are common and can lead to significant variations in indices: *e.g.* sampling may be carried out indoors or outdoors only, or at both locations; the presence of cryptic breeding sites may lead to under-sampling or complete omission of certain sites; failure to distinguish *Aedes aegypti/albopictus* from other common mosquito species, or from each other, may lead to overestimates. Little is known about the relationship between differing proportions of the various sampled larval instars and the accuracy of these data as proxy measures of adult mosquito abundance [17]. Finally, although ovitraps (water-filled pots in which *Aedes aegypti* lay their eggs) are widely used as a simple sampling tool, Focks [15] showed very convincingly that their reliability is limited to indicating vector presence or absence.

Despite these doubts, many dengue control authorities worldwide routinely collect vector population data based on these indices, although the mathematical relationship between any of the indices and dengue transmission is far from clear. Thresholds indicating dengue outbreak risk for House and the Breteau indices (HI = 1%, BI = 5) have been used for many years [18], [19], even though these values were developed for yellow fever many decades earlier. Simple thresholds may be valid in some situations [20], but a universal critical threshold applicable across many contexts, has never been determined for dengue. In pursuing the goal of identifying dengue thresholds, Scott & Morrison [21] defined the fundamental knowledge gaps as: 1) what is an acceptable level of dengue risk?; 2) what are the mosquito densities necessary to achieve that goal?; 3) what is the best way to measure entomological risk?; 4) at what geographic scale are the components of dengue transmission important? While a number of mathematical models have explored the value of thresholds or rates of change in the vector population for the prediction of dengue outbreaks [22], [23], these knowledge gaps remain and continue to hinder progress [24]. For convenience, dengue outbreaks are often defined as periods when dengue incidence is equivalent to the mean plus 2 standard deviations during the same month of the previous year [25].

Effective dengue surveillance and early warning systems, using information from multiple epidemiological sources, are an important goal for numerous countries worldwide. To determine the value of vector surveillance for such systems, the findings of a systematic review examining the evidence for a relationship between mosquito indices and dengue cases are reported here.

## Methods

### Objectives

The aim of the study was to evaluate the potential value of vector or entomological survey data for dengue surveillance by examining the evidence from studies that investigated quantitatively the relationship between vector indices and dengue cases. The specific objectives were:

1. To identify vector surveillance methods and indices used for the routine monitoring of *Aedes aegypti* or *Aedes albopictus* populations in any geographic location.
2. To examine how entomological indices correlated with dengue incidence.
3. To examine the effectiveness or accuracy of vector surveillance in predicting dengue outbreaks and consider how this might be improved.

### Search Strategy

A review protocol was established and agreed upon by all authors. Guidelines from the Cochrane Handbook for Systematic Reviews and the PRISMA Group were followed as standard methodologies [26], [27]. The databases WHOLIS, PubMed, EMBASE, LILACS and Web of Science were searched using the Medical Subject Heading (MeSH) “dengue” followed by the Boolean operator “and” combined with one of each of the following ‘free text’ terms in succession: ‘entomological surveillance’, ‘oviposition trap’, ‘house index’, ‘container index’, ‘Breteau index’, ‘pupal index’, ‘pupal survey’, ‘adult collection’, ‘sticky trap’, ‘aspirator collection’, ‘resting collection’, ‘landing collection’, ‘vector density’. The reference list of each of the included studies was also searched, and “grey literature” was sought by communication with authors for cited unpublished documents.

Results were collated in EndNote (EndNote X5, Build 7473) where abstracts were reviewed in accordance with agreed inclusion and exclusion criteria. Full text review was completed using ‘Papers’ (Papers 2, version 2.2.10). No limits were placed on year of publication, language or location.

### Inclusion and Exclusion Criteria

The criteria for inclusion or exclusion of individual studies were set in advance (Table 1) and were used to assess each abstract and/or the full text.

**Table 1:** Criteria for inclusion or exclusion of studies.

Inclusion Criteria	Exclusion Criteria
Any study where entomological surveillance of *Aedes* spp. was undertaken for >3 months (or for the duration of a dengue outbreak) in conjunction with number of reported dengue cases	Studies with only one outcome of interest (entomological surveillance OR dengue cases);
Any study type with all empirical data gathered within the same time period	Opinion papers; review articles; retrospective analyses comparing data generated at different time points
Confirmed and/or probable dengue cases identified using WHO standard case definition and/or serology	Qualitative dengue reports

### Definitions

The following definition was used for the term ‘vector surveillance’: “Any ongoing surveillance of entomological indices, including larval indices (House Index (HI), Container Index (CI), Breteau Index (BI)), pupal indices (Pupal Productivity Index (PPI) and other variations), oviposition trap data and data from adult mosquito collections (methods include sticky, traps, CO_2_, odor-baited, visual or other traps, resting catches, human landing catches), used in relation to dengue outbreak/control.”

### Quality Assessment

Given the strict nature of the inclusion criteria, study design was assessed at the data extraction stage using the Quality Assessment Tool for Quantitative Studies (QATQS) [28]. QATQS provides a recognized standardized method to assess study quality by assigning scores based on possible selection bias, study design, confounders, data collection methods, intervention integrity and statistical analyses. This ensured each study could be ranked qualitatively. The study design classes were intervention, case-control and longitudinal. If clarification was required, authors were contacted for any missing data or information.

### Data Extraction and Assessment

The information extracted included first author, year of publication, year of study, population size, study design, indices and case definitions, study objectives, duration of study, frequency of data collection, results and conclusions (as viewed by all reviewers; Table S1). A table of bias was created to help identify the strengths and weaknesses of each study (Table S2).

### Ethics Statement

No ethical review was required for this systematic literature review.

## Results

**Figure 1:**
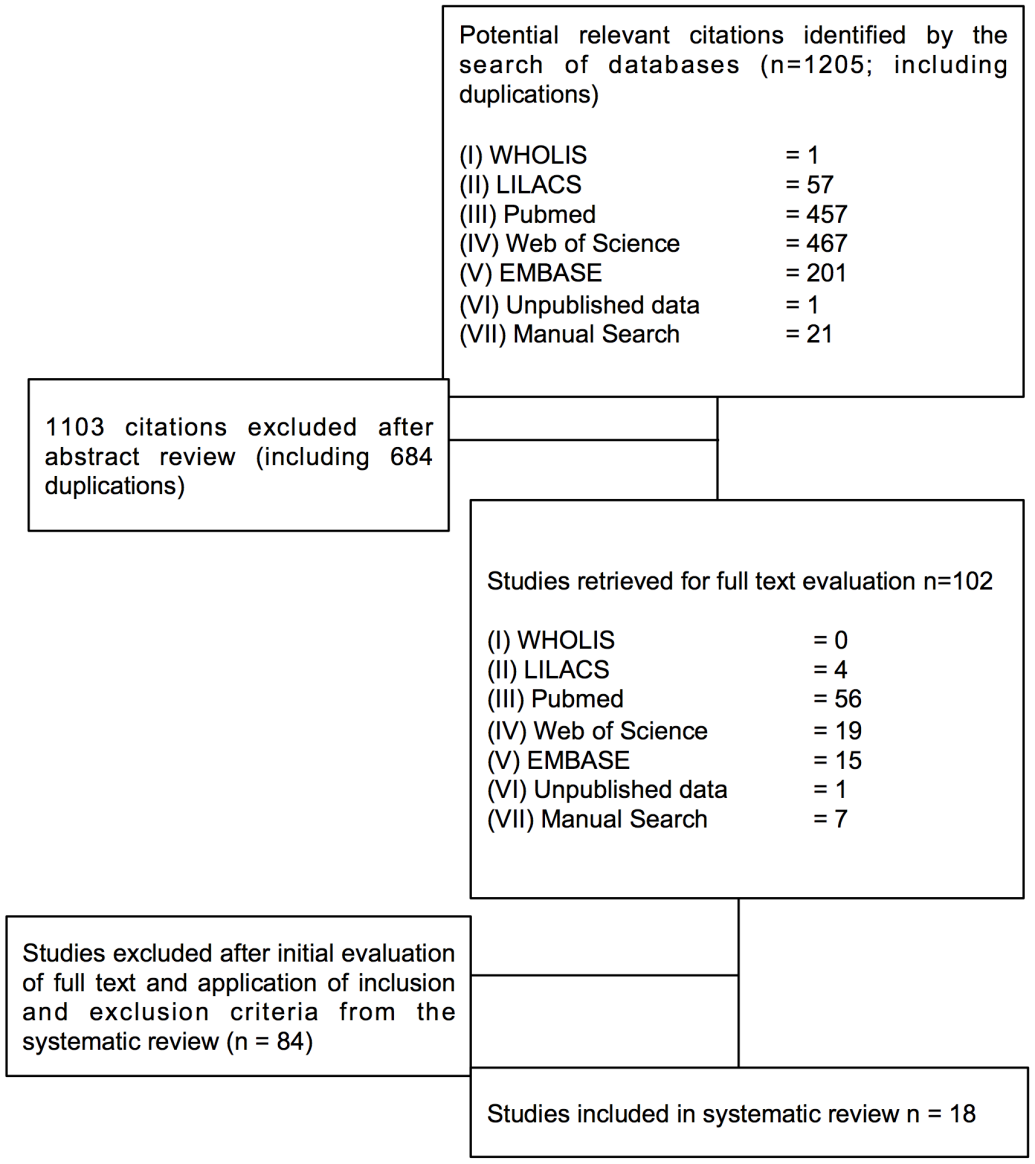
Search Tree. Diagram of searches performed and the number of articles returned and examined at each stage.

A total of 1205 potentially relevant studies were identified in the database search. After reviewing abstracts, 102 were selected and retrieved for full text evaluation, of which 18 were considered to have satisfied all inclusion and exclusion criteria and explored in detail (Figure 1) [20], [29]–[45].

Regarding the 84 studies excluded, the most common reasons for exclusion were: study duration less than 3 months (22 studies); absence of a reliable dengue case definition (21 studies); use of datasets that did not correspond temporally or spatially (19 studies). Note that although such dislocated spatial comparisons were not captured by the exclusion criteria originally defined (simply because it had not been expected), exclusion at this point was considered to be valid. Other reasons for exclusion were: measurement of only one outcome (*i.e.* vector or dengue cases only: 9 studies); opinion or review articles (8 studies); use of incomplete datasets – where only ‘selected’ portions of all of the data available during the study period were used (5 studies). Again, although the latter reason was not captured by the original criteria, exclusion of studies where this occurred was considered to be valid. Full details of the 18 studies reviewed are summarised in the supporting data files (Checklist S1, Table S1, Table S2).

The origin of the data used in analyses differed between studies. Some generated novel data as an integral part of the study, thus ensuring complete or independent control over the quality of the data obtained, while others obtained existing or retrospective data from external sources, including local surveillance data (*e.g.* local government records, private companies, hospitals or health centers, independent physicians and self-reported data). Twelve studies generated vector data [30]–[32], [34]–[40], [42]–[44], five generated dengue case data [29], [30], [35], [36], [38], four of which generated both vector and dengue case data [30], [35], [36], [38].

### Study Design

Fourteen studies were longitudinal, two were case-control, one was an ecological study (as defined by the unit of analysis) and one was a vector control intervention. Applying QATQS [28], fifteen studies [20], [30]–[33], [35]–[41], [43]–[45] scored 3 (defined as a weak study), two studies [29], [42] scored 2 (a moderate study design) and one study [34] scored 1 (a strong study design)(Annex 2). In the latter study, Chadee and colleagues [34] used controls matched on age and sex from a neighboring community, although the report did not state whether or not this process was randomized.

### Vector Sampling

Details of the sampling protocols used in each study are shown in Table 2. Eleven of eighteen studies generated indices for immature stages of the vector and collected combined larval and pupal numbers to calculate either the CI, HI or BI [20], [29]–[32], [34], [35], [37], [40], [42], [43]. One of these [37] combined *Ae. aegypti* and *Ae. albopictus* data. Four studies sampled only larvae [33], [36], [44], [45].

**Table 2:** Details of vector sampling methods used and correlation of vector indices with dengue transmission in the studies reviewed.

Ref. Number	Study	Immature Vector Indices					Adult mosquitoes sampled	Egg (ovitrap) sampled	Location		Sample spatial unit	Significant (*p*≤0.05) increase in vector indices recorded during dengue transmission
		CI	HI	BI	BI_max_	Pupal Index			Indoor	Indoor + Outdoor		
**20**	**Sanchez ** ***et al.*** **, 2010.**			**▪**	**▪**		**∼**		✓		**Block; N'hood**	**∼**
**29**	**Sanchez ** ***et al.*** **, 2006.**	**▪**	**▪**	**▪**	**▪**		**+**		✓		**Block; N'hood**	**+**
**30**	**Chadee, 2009**	**▪**	**▪**	**▪**		**pupae/person**	**+**			✓	**Premise**	**+**
**31**	**Pham ** ***et al.*** **, 2011**	**▪**	**▪**	**▪**			**+**		✓		**Premise**	**+**
**32**	**Gurtler ** ***et al.*** **, 2009**		**▪**	**▪**			**∼**		✓		**N'hood; City**	**∼**
**33**	**Katyal ** ***et al.*** **, 2003**	**⧫**	**⧫**	**⧫**			**∼**					**∼**
**34**	**Chadee ** ***et al.*** **, 2005**			**▪**			**+/−**			✓	Premise*	**+/−**
**35**	**Romero-Vivas & Falconar, 2005**	**▪**	**▪**	**▪**		**pupae/premise**	**−**			✓	**Premise**	**−**
**36**	**Foo ** ***et al.*** **, 1985**		**⧫**	**⧫**			**−**		✓		**Premise**	**−**
**37**	**Sulaiman ** ***et al.*** **, 1996**		**▪⊙**	**▪⊙**			**+/−**		✓		**City zone**	**+/−**
**38**	**Honorio ** ***et al.*** **, 2009**						**−**	✓			**Premise**	**−**
**39**	**Rubio-Palis ** ***et al.*** **, 2011**						**+**		✓		**Premise**	**+**
**40**	**Lin & Wen, 2011**			**▪**	**▪**		**+/−**			✓	**District; Min admin unit**	**+/−**
**41**	**Chaikoolvatana ** ***et al.*** **, 2007**	**▪**	**▪**	**▪**			**∼**			✓	**Village**	**∼**
**42**	**Chadee ** ***et al.*** **, 2007**		**▪**	**▪**			**∼**			✓	**County**	**∼**
**43**	**Correa ** ***et al.*** **, 2005**						**+/−**				**District; Trial area**	**+/−**
**44**	**Fernandez ** ***et al.*** **, 2005**	**⧫**	**⧫**	**⧫**			**+/−**				**Premise**	**+/−**
**45**	**Arboleda ** ***et al.*** **, 2012**			**⧫**			**−**				**0.25 km^2^**	**−**

All studies reported *Ae. aegypti* alone unless indicated otherwise. HI = House Index (% houses with larvae and/or pupae); CI = Container index (% water-holding containers with larvae or pupae); BI = Breteau index (no. positive containers per 100 houses inspected); BI_max_ is defined as the highest or ‘maximum’ block level BI in a neighborhood; Pupal index = pupae per person/premise defined as no. pupae divided by the number of residents/premises.

Immature vector samples are denoted as: ⧫ larvae only; ▪ larvae and pupae; ⊙ *Aedes aegypti* & *Aedes albopictus* combined.

Cells marked ✓ indicate the sampling activity was done.

The Sample spatial unit referred to as ‘P*’ is the ‘premise with cardinal points index’ [34]; ‘N'hood’ = neighborhood.

In the right-hand column, the reported association between vector indices and dengue cases is classed as: ‘**+**’ positive association; ‘**−**’ no association; ‘**+ −**’ ambiguous association; ‘**∼**’ inconclusive or weak association.

Absence of any entries in cells indicates no data or information was reported.

Thirteen studies reported the location of the immature stage mosquito samples: six studies sampled both indoor and outdoor containers [30], [34], [35], [40]–[42], while seven searched indoor containers only [20], [29], [31], [32], [36], [37], [39]. Thus, where reported, all studies included indoor sampling.

Pupal indices were reported in two studies [20], [35]. Adult mosquitoes were sampled in three studies [38], [39], [43].

### Relationship between Entomological Indices and Dengue Cases

Thirteen studies examined the association between entomological indices and dengue, using a range of different statistical approaches. Seven studies calculated regression coefficients [36], [37], [39], [40], [43]–[45], two calculated rate ratios [31], [38], one calculated odds ratios [29] and two calculated the G-test for significance [32], [36]. One study used only specificity, sensitivity and positive and negative predictive values [20].

The spatial unit of analysis, an important consideration in dengue epidemiology (see Discussion) varied considerably across studies, with units ranging from individual houses, housing blocks and clusters to neighborhoods and even large municipalities (Table 2).

Four studies reported statistically significant positive relationships between entomological indices and dengue incidence [29]–[31], [39]. Of these, only one sampled adult mosquitoes (33% of those studies that sampled adults) [39] while the remainder sampled immature stage mosquitoes (20% of all those that sampled immatures) [29], [30], [31](Table 2). These are discussed in detail here.

### Evidence for Positive Correlation between Vector Indices and Dengue Cases

Sanchez (2006) [29] conducted a case control study using two geographical units for analysis, blocks (units of approximately 50 houses) and neighborhoods (each containing approximately 9 blocks). Any block or neighborhood with at least 1 confirmed case was considered positive, while a control was defined as a block or neighborhood without confirmed cases. HI and BI mean values were “consistently, substantially and significantly higher” in blocks with dengue cases compared with control units. An odds ratio (OR) of 3.49 (p<0.05) for dengue transmission was associated with the presence of a single positive container in a block; fifteen of the seventeen dengue cases recorded lived in a neighborhood where at least 1 block had a BI>4.

In Trinidad, Chadee (2009) [30] compared retrospective routine entomological household data with concurrent entomological data taken from confirmed dengue households, using a cardinal points approach (*i.e.* the ‘index’ house plus the four adjacent houses at its cardinal points). Chadee found that significantly more (P<0.001) immatures were collected during dengue case investigations than during the routine inspection and treatment cycles. The report also stated that pupae per person indices were higher and significantly more adults emerged (as a function of total pupae count collected from household containers) at locations where dengue was confirmed at the index house, compared with routine investigations.

Pham *et al.* [31], examined monthly dengue case data, vector larval indices and meteorological data from central Vietnam, between 2004 and 2008. They found significant associations between all entomological indices and dengue cases by univariate analysis but only the HI and “household mosquito index” (not defined in the paper), temperature and rainfall were significant after multivariate analysis.

In Venezuela, Rubio-Palis *et al.* [39] used a simple regression analysis to investigate correlations between vector indices, climatic variables and dengue incidence for the period 1997–2005. Analyses indicated a significant relationship (R^2^ = 0.9369) between the numbers of dengue cases, *Ae. aegypti* abundance (both immatures and adults) and rainfall. Acknowledging the retrospective nature of the study, the authors expressed caution in the predictive value of the findings. Moreover, another limitation was that entomological data were derived only from actual homes and neighbouring houses of confirmed dengue cases but no data were collected from ‘control’ houses.

### Value of Vector Indices for Advance Warning of Dengue Outbreaks

Within these four studies was some additional evidence that observed changes in vector indices might be useful for the prediction of impending dengue transmission or outbreaks. In Cuba, Sanchez (2006) [29] reported that blocks with BI_max_ (defined as the highest or ‘maximum’ block level BI in a neighborhood) values greater than 4 were significantly more likely to record positive cases in the following month, and had a 3–5 times greater dengue risk in comparison with control blocks. The report concluded that BI_max_>4 and neighborhood BI>1 during the preceding 2 months provided “good predictive discrimination”. In northern Venezuela Rubio-Palis *et al.* [39] found the most significant correlation between rainfall levels and the appearance of dengue cases two months later, indicating that the magnitude of outbreaks might be predictable to some extent following periods of rainfall. Pham *et al.* [31] confirmed an association between dengue transmission and periods of higher rainfall and mosquito abundance in the central highlands of Vietnam, but did not indicate whether this could be used in advance of transmission as a predictive tool.

### Unreliable or Absence of Correlation between Vector Indices and Dengue Cases

A further five studies [34], reported ambiguous evidence of associations, both positive and negative, between entomological data and dengue cases. In Belo Horizonte, Correa *et al.* [43] found a 5–7 fold increase in mean monthly dengue incidence where the ‘infestation rate’ (defined as house index) was “between 1.33% and 2.76% and equal to or higher than 2.77% when compared to areas showing 0.45% or less”, although it was unclear whether or not this was statistically significant. They reported a moderate but significant correlation between adult *Aedes spp.* infestation rates and numbers of dengue cases (R = 0.67) even though HI and dengue cases were only weakly correlated (R = 0.25 at the municipal level; R = 0.21 and R = 0.14 at the district and village level). Sulaiman *et al.* [37] reported a significant correlation between BI and HI and dengue cases in certain areas of Kuala Lumpur, but not in others. In Trinidad, Chadee *et al.* [34] found that 75% of DHF cases were located in areas where BI was greater than 10, although BI and dengue infections were rarely correlated. An additional two studies reported either very low correlations between vector indices and dengue [44], or utilized highly variable inter-annual data precluding such analyses [40].

Four studies, from Malaysia [36], Brazil [35] and Colombia [35], [45] found no statistically significant relationships between entomological indices and dengue cases. Foo *et al.* [36] observed a positive but non-significant association between dengue cases and HI and BI, which they suggested may have been influenced by the small sample size, the presence of *Ae. albopictus* and socio-demographic factors. Honorio *et al.* [38] found no significant associations between recent dengue cases and *Ae. aegypti* densities and proposed that infections received outside the home were responsible. In Colombia, Romero-Vivas and Falconar [35] reported distinct positive temporal correlations between the larval density index and pupal density index (p<0.005) and a negative association between the larval density index and egg density index (p<0.01); however, they found no correlation between any of the larval, pupal or adult indices with either rainfall or dengue-like cases. The spatial model of Arboleda *et al.* found no indication that the BI was in any way correlated with the dengue cases or those areas predicted as ‘suitable’ [45].

In the remaining studies [20], [32], [33], [41], [42] a variety of mixed, inconclusive or weak associations were reported. Gurtler *et al.* conducted analyses on the effect of a given intervention on mosquito indices but not on dengue cases [32]. Although Katyal *et al.* [33] did not present any statistical analysis, they reported the observation that over a five year period, a fall in cases was visually correlated with a fall in indices. However, they conceded that “an increasing trend of cases was observed [in 2001] in spite of a further declining HI trend”, and concluded that HI had no predictive value at the ‘macro’ level. Despite the absence of statistical analysis, Chaikoolvatana *et al.* [41] reported a suggestive link between dengue haemorrhagic fever (DHF) during peak annual rainfall months and high abundance of mosquitoes. Chadee *et al.* observed ambiguous associations, with BI partially correlating with dengue fever cases for two out of three years [42]. As in their earlier study at the same Cuban location [29], Sanchez et al [20] reported that while BI_max_≥4 was a useful predictor for outbreaks at the block level, sensitivity during outbreaks ranged between 62% and 81.8% and specificity between 71.9% and 78.1%.

### Use of Vector Indices as Transmission Thresholds

**Figure 2:**
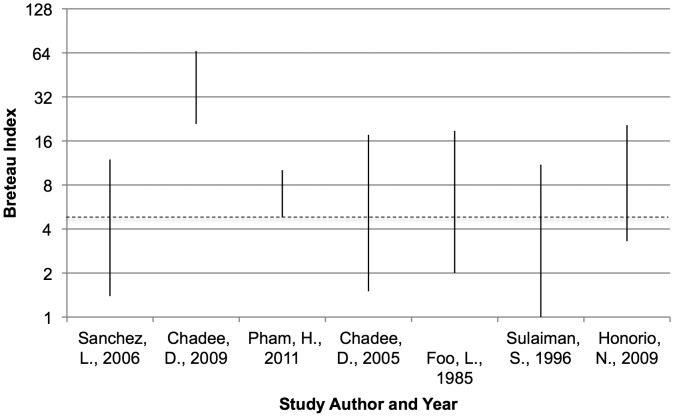
Range of Breteau indexes reported during dengue transmission. Dotted line indicates a BI value of 5, which has been considered a transmission threshold for dengue [21], [45], [52]. Note: Includes all data where available, whether statistically significant or insignificant.

The Breteau Index (BI) was used as an outcome measure in seven studies [29]–[31], [34], [36]–[38] and BI_max_ threshold was considered in three (Table 2) [20], [29], [40]. Here, BI values ranged from 1 to 66 during periods when dengue transmission was recorded (Figure 2). In other studies, both recent [46] and historic [47], dengue transmission was recorded when BI values were lower than the widely accepted transmission threshold of 5. Notably, in a study in Trinidad, ‘high’ transmission (25–40 cases for 75% of sample ‘cycles’) took place in areas with relatively ‘low’ abundance (∼BI<5) while, conversely, a consistently higher BI of 5.4 in neighbouring areas did not result in dengue cases [34]. In Rio de Janeiro, the BI did not correlate with dengue incidence and transmission occurred in association with a wide range of BI levels (range 3.30–20.51) [38].

## Discussion

With worldwide dengue transmission levels at an all time high, predicting dengue outbreaks in advance of their occurrence or identifying specific locations where outbreak risks are highest is of critical importance. This review considered the evidence that changes in vector populations can be correlated with dengue virus transmission and whether or not monitoring fluctuations in vector indices might be employed to provide reliable advance warning of impending dengue outbreaks.

Eighteen studies that had the potential to provide evidence of any association between vector indices and dengue incidence were identified and examined. Notably, only 4 studies utilized new data on both vector indices and dengue cases collected *de novo* as an integral part of the study. More common was a reliance on local government-level records for the dengue case data, a practice that potentially introduces error or bias for number of reasons. First, hospital reports are prone to selection bias, as asymptomatic/inapparent infections may not be recorded and the actual number of cases may have been significantly underreported. Second, there can be a considerable delay between the times of onset of infection and reporting which, if the infection date is not calculated, would result in a temporal mismatch of vector and case data. Third, differences between the geographic location of the vector and dengue case data, or between the spatial units from which each was originally calculated, would result in a geographic mismatch or mask potential relationships, respectively.

The latter point is of particular significance not only from the point of view of these studies, but also when considering the design of future investigations. A growing body of evidence indicates that the distribution of dengue cases typically is highly clustered in both time and space. In various studies, post-dating those reviewed, the size of such clusters ranged from 800 m [48] to less than 100 m [49]. The effective area of such key ‘pockets’ or ‘hotspots’ is likely to be determined by dispersal of the vector [49], [50] which itself can vary over time [51], and is influenced by house density [52] and by human movement within and beyond the infection cluster [53]. Consequently, in studies attempting to correlate vector indices with dengue transmission, and where the geographical unit is too large, high vector densities in key dengue hotspots might be diluted by inclusion of neighboring areas with low densities, thus masking any true relationships [see 38].

Indeed, human movement potentially confounds dengue vector data that derive from residential areas alone as increasingly, evidence indicates that only a proportion of dengue infections are transmitted in the individual's own home, with many infections (possibly the majority) resulting from bites by virus-infected mosquitoes at other houses, schools, workplaces or numerous locations remote from the home [53], [54]. Clearly, this presents a serious challenge when considering the use of vector data for surveillance and highlights a need for inclusion of data from public locations [55] in addition to residential areas, in any surveillance program.

Returning to the studies examined in this review, the fact that there was no clear indication of any consistent association between vector indices and dengue cases is not unexpected, given the diverse and mostly weak study designs. One study found there was no apparent increase in vector indices coinciding with what was the largest increase in dengue fever cases of all areas studied [40], while in another, dengue transmission remained low despite exceptionally high vector indices [44]. In studies where correlations were calculated for HI, BI and dengue cases, regression coefficients ranged from weak/moderate non-significant (R = 0.43 and R = 0.35 respectively; p>0.05) [38], to moderate significant associations (R = 0.61 and R = 0.60 respectively, but only in the urban centre; p<0.05) [37].

Only two studies calculated pupal indices, even though fifteen of the eighteen studies reviewed were published more than three years after WHO acknowledged that the traditional *Stegomyia* indices were inadequate for the measurement of dengue vector abundance [56]. In the two studies included in this review that calculated pupal indices, only one reported increases in the pupal index, but its relationship with dengue cases was not statistically significant, possibly due to the low numbers of pupae recorded [30], [35]. A major problem with pupal surveys is the difficulty in locating breeding sites and the potential existence of important or key but cryptic breeding sites (*e.g.* overhead tanks on houses or underground water reserves such as sewers or wells) that may harbor significant proportions of the vector population [57], [58].

Clearly, calculation of adult female *Aedes aegypti* indices is the most direct measure of exposure to dengue transmission [15]. Of the four studies reviewed that reported some correlation between vector indices and dengue cases, two [31], [39] recorded adult vector data. The adult population of *Aedes aegypti* is rarely sampled, partly due to the erroneous but commonly held belief that carrying out such sampling is time-consuming, difficult or expensive [59].

Sampling adult female *Aedes aegypti* is a relatively simple task, though it can be limited by the fact that mosquito numbers often remain low during outbreaks [60]. Nonetheless, it is possible to aim to sample adult mosquitoes as a routine procedure with minimal additional training and resources. A number of novel sampling devices [61]–[63] offer the potential to monitor vectors during outbreaks [64] and at the spatial scale required to accurately sample populations of *Ae. aegypti* [65]. Simple affordable low-tech tools that enable localized sampling of adult *Ae. aegypti* and other mosquito vectors are available, with initial studies demonstrating their ease and effectiveness in comparison with older methods [66], [67]. In Brazil, routine sampling of *Ae. aegypti* adults with gravid traps deployed at relatively low densities was used to identify high risk localities which were then targeted for vector control [68], [69]. This ‘Intelligent Dengue Monitoring’ system was reported to have prevented over 27,000 dengue cases over two ‘dengue seasons’ between 2009 and 2011 with considerable reductions in cost burden to the communities where it was deployed [70].

None of the studies reported on viral infection rates in the vector. This perhaps is not surprising given that techniques suitable for application in routine surveillance, such as PCR or NS1, have not been available until recently, that vector infection rates with dengue virus are of the order of 1% even in areas where transmission is ongoing [64], [70]–[72] and the cost of running the large numbers of tests to detect meaningful infection levels could be considered prohibitive for many authorities. Nonetheless, routine screening for dengue virus of trapped adult female *Aedes aegypti* is possible and has been incorporated into the routine surveillance program in Belo Horizonte, Brazil [73]. The relative low dispersal rates of *Ae. aegypti* as compared with the high mobility of humans as they commute daily from the home to the workplace, school, etc., means that virus-infection rates in the vector potentially could provide an accurate or epidemiologically valid indicator of dengue risk in any particular locality, thus informing vector control. Clearly, elucidating the relative value of such an index would require substantial research investment, while integrating it into routine surveillance programmes would demand significant sustained investment, but the importance of metrics like the sporozoite or entomological inoculation rates used in malaria epidemiology [59] already indicate the potential.

This review has also demonstrated the unreliability of accepted vector thresholds for dengue transmission. A number of studies reported dengue transmission at BI levels below the currently accepted threshold of 5 (Figure 2) [29], [34], [36]–[38] or when the HI was below 1% [74], [75]. Elsewhere, Focks proposed a pupal productivity index of 0.25 as a threshold for dengue transmission in Honduras [76], yet in Brazil dengue transmission occurred at PPI levels of 0.15 [58]. While the desire for a single globally applicable transmission threshold is understandable, it seems unlikely that such a threshold exists, given the variety and complexity of other parameters that potentially influence the risk of outbreaks today [19], [77], [78]. Chadee concluded in 2009 that dengue transmission occurs, not at a fixed entomologic figure/quantity but rather at a variable level based on numerous factors including seroprevalence, mosquito density and climate [30]. It is becoming increasingly apparent that thresholds differ at different locations and in different contexts, and while they must be calculated independently at each location [19], [79]. Moreover, empirically defining thresholds, which must be expected to be dynamic, rising and falling as the susceptibility of the local population changes, will require comprehensive prospective, longitudinal vector studies [80], with simultaneous monitoring of the relationship between *Ae. aegypti* population densities and dengue virus transmission in a spatially relevant human cohort.

### Study Limitations

In spite of reference searches and use of grey literature, publication bias will likely remain given the very nature of a systematic review. However, we also sought to further limit the effect of publication bias by placing no restriction on language, and those languages encountered were: English, French, Portuguese, Spanish and Chinese.

Additionally, one should be cautious when interpreting these data due to the study design of the 18 articles. As defined by QATAS assessment methods, study design was often weak (15 studies), meaning that studies were prone to bias and confounding factors, which may have skewed some of the reported associations. In addition, most (n = 13) studies relied on dengue case data from external sources, rather than obtaining study-generated data. With the exception of vector sampling and generation of vector index, there were few similarities in the approaches across the different studies.

### Conclusions and Recommendations

Despite the widespread practice of collecting vector population data, the review has revealed that very few rigorous studies have been undertaken to determine the relationship between vector abundance and dengue transmission; of those that have been published, few provide tangible evidence of such a relationship, and therefore it is not possible to draw a firm conclusion. After decades of vector surveillance in many countries and considering the magnitude of the dengue threat today both in those and other countries that have recently experienced major dengue outbreaks, this is disappointing. Yet it is also indicative of the lack of basic knowledge of dengue epidemiology, in particular with regard to transmission. Clearly, this is a major knowledge gap that requires attention with a degree of urgency and the following research priorities are recommended:

- The relationship between vector population abundance and dengue transmission remains unknown and should be quantified. Studies should aim to collect new vector and clinical datasets carefully matched temporally and spatially. Given that epidemiology will vary considerably between different contexts and geographical localities, multiple locations should be investigated.
- The ideal and most powerful approach would be for a series of coordinated studies, to be carried out in multiple locations worldwide, as exemplified by recent examples [80]. To facilitate such studies, and ensure higher power in individual and combined datasets, the development of a standardized study design and protocols is a priority.
- Individual locations are also strongly encouraged to investigate the relationship independently. Many dengue-affected areas (cities, districts or similar spatial units) are likely to have substantial historic vector and dengue data that potentially may be suitable for appropriate analysis.
- Spatial heterogeneity and transmission at sites other than the home must be considered and carefully incorporated into any study design.
- The utilization of single global values of the Breteau (BI) or other vector indices as thresholds for dengue transmission is unreliable and is not recommended.
- While the need for a standardized reliable definition of a dengue outbreak has already been stated elsewhere [81], research into the relationship between vector abundance and dengue transmission should endeavor to develop a similar approach to defining reliable locality-specific vector population indices (*e.g.* thresholds, rates of increase, etc.) for use as early warning signals for impending increases in dengue transmission.
- Adoption of adult dengue vector sampling by all vector surveillance programs is urged. Various new trapping methods, as well as a simple resting catch approach, should be evaluated.
- Relationship between larval, pupal and adult stages of the vector population and the factors influencing adult emergence rates remain poorly understood. The paucity of fundamental knowledge of the ecology of mosquito vectors generally and the need for basic studies has been advocated elsewhere [82], [83] and is true for *Ae. aegypti* and *Ae. albopictus*. A greater understanding of the ecology of dengue vectors is essential.

In the absence of definitive evidence that dengue vector surveillance data can contribute to the prediction of dengue outbreaks, it might be tempting to consider abandoning the practice altogether. However, this would be a rash and premature judgment. At the very least, this systematic review has demonstrated that the potential of vector surveillance data has not yet been evaluated. Indeed, its full potential will not be apparent until its contribution to a complete predictive model incorporating all other covariates influencing dengue epidemiology have been considered. That will not be possible until multiple high quality studies investigating the relationship between vector populations and dengue transmission have been carried out.

## Supporting Information

Checklist S1 PRISMA checklist.
(DOC)

Table S1 Data extraction table summary for reviewed studies.
(XLSX)

Table S2 Assessment of the validity of reviewed studies: Table of bias and QATQS (Quality Assessment Tool for Quantitative Studies) rating for each study.
(XLSX)
